# In Search of Better Peptide-(Derived from PD-L2)-Based Immune Checkpoint Inhibitors

**DOI:** 10.3390/biom14050597

**Published:** 2024-05-18

**Authors:** Boris Klebansky, Marina Backer, Vitaliy Gorbatyuk, Olga Vinogradova, Joseph Backer

**Affiliations:** 1 BioPredict Inc., 4 Adele Ave., Demarest, NY 07627, USA; 2 SibTech Inc., 115A Commerce Drive, Brookfield, CT 06804, USA; 3 Center for Open Research Resources & Equipment, University of Connecticut, Storrs, CT 06269-3060, USA; 4 Department of Pharmaceutical Sciences, School of Pharmacy, University of Connecticut, Storrs, CT 06269-3092, USA

**Keywords:** PD-1, PD-L1, PD-L2, inhibitors, modeling, FRET, NMR

## Abstract

Current anti-cancer immune checkpoint therapy relies on antibodies that primarily target the PD-1/PD-L1(-L2) negative regulatory pathway. Although very successful in some cases for certain cancers, these antibodies do not help most patients who, presumably, should benefit from this type of therapy. Therefore, an unmet clinical need for novel, more effective drugs targeting immune checkpoints remains. We have developed a series of high-potency peptide inhibitors interfering with PD-1/PD-L1(-L2) protein–protein interaction. Our best peptide inhibitors are 12 and 14 amino acids long and show sub-micromolar IC_50_ inhibitory activity in the in vitro assay. The positioning of the peptides within the PD-1 binding site is explored by extensive modeling. It is further supported by 2D NMR studies of PD-1/peptide complexes. These results reflect substantial progress in the development of immune checkpoint inhibitors using peptidomimetics.

## 1. Introduction

PD-1, programmed cell death protein 1, is known to inhibit immune activation upon interaction with its ligands, PD-L1 or PD-L2. Antibody-based immune checkpoint therapy targeting PD-1/PD-L1(-L2) interactions is validated and demonstrates the possibility of a significant, even dramatic, therapeutic improvement in some patients. Despite its low efficiency for the majority of patients and a high cost of treatment, there are currently more than four thousand trials targeting PD-1 and PD-L1 with various combinations of antibodies and other treatment modalities (clinicaltrials.gov accessed 11 October 2023). The relatively low efficacy of checkpoint antibodies is due, in part, to their complex PK/PD (pharmacokinetic/pharmacodynamic) characteristics [1], significant systemic toxicity [2,3], and induced tumor resistance mechanisms [3,4]. Furthermore, there are well-known general limitations of antibodies as drugs [5,6], such as poor diffusion and tumor permeability, irreversible binding to the first encountered antigen at the tumor periphery, and promiscuous interactions with Fcγ receptors on various cells, including tumor-associated macrophages, which “steal and sequester” antibodies from their tumor targets [7]. Thus, there is an unmet clinical need for new, efficient, less expensive immune checkpoint inhibitors.

Several factors support our rationale for the development of peptide molecule inhibitors of PD-1/PD-L1(-L2) interactions, as opposed to small molecule inhibitors. First, the analysis of relevant crystal structures indicates the lack of suitable pockets for small molecules on the rather flat protein–protein interfaces of PD-1 and PD-L1(-L2) [8,9,10]. Second, the originally reported small molecule inhibitors did not show impressive results [11,12,13,14]. In fact, extensive biophysical and tissue culture evaluation of the leading compounds from Bristol Myers Squibb and Aurigene Discovery Technologies revealed that they do not directly block PD-1/PD-L1 interactions and are highly cytotoxic [15]. More recent studies, however, have confirmed that small molecule inhibitors can, indeed, cause internalization of PD-L1 [16], followed by induced stimulation-dependent cytokine production in primary human immune cells [17]. Lastly, we reasoned that designing a small peptidomimetic inhibitor would be more feasible once a potent peptide inhibitor has been found. 

The design of the first published peptide inhibitors targeting PD-L1 was guided by the binding epitopes of anti-PD-L1 antibodies [18,19]. Unfortunately, in that study, the IC_50_ values of two lead peptides in tissue culture assay were only in a high sub-micromolar range [19]. We reasoned that using peptide fragments based on protein–protein interfaces of PD-1 or PD-L1(-L2) may provide an effective approach to inhibiting the formation of PD-1/PD-L1(-L2) complexes. Interestingly, judging by the interactions between recombinant ectodomains of PD-1 and PD-L1, the association between these proteins is relatively weak, with the reported K_d_ values in a high sub-micromolar to a micromolar range [8,10,18]. However, mutational and modeling studies of human PD-1 indicated that the affinity could be significantly improved [20,21,22,23], suggesting that re-engineering native sequences may lead to high-affinity peptide inhibitors. Indeed, several groups reported peptide inhibitors based either on the fragments of PD-1 [24,25,26] or PD-L1 [27,28] or derived from the panning of the phage display libraries [29,30]. Although these studies were informed by the high-resolution crystal structure of the human PD-1/PD-L1 complex [9], so far, reported peptides had, at best, low micromolar affinity to target proteins. The most recent publication [31], where authors explored PD-1-derived peptides to inhibit the PD-1/PD-L1 complex, still presents K_d_ values at micromolar range. Taken together, the development of peptide-based inhibitors of the PD-1/PD-L1(-L2) immune checkpoint is at the very early stage, and we see exciting opportunities for significant advances in this area.

The selection of PD-1 as a target is determined by a superior clinical effect achieved with anti-PD-1 antibodies compared to those directed to PD-L1 [32]. Such a difference could be due to a potential tumor cell escape via switching from PD-L1- to PD-L2-mediated engagement of PD-1 [32]. This reasoning would remain valid for PD-L1 peptide inhibitors.

We focused on targeting PD-1 with peptides composed of 12–14 amino acids derived from the structure of PD-L1(-L2) β-hairpins, a central part of the protein-protein interface in complexes of PD-1 with PD-L1(-L2). We designed several series of peptides for enhanced binding via the formation of the induced fit of the specific PD-1 region involved in the protein–protein interface and screened those peptides in a tissue culture assay that measures PD-1/PD-L1 binding. As a result of our screening, we obtained several peptide inhibitors with low sub-micromolar IC_50_ values. 

## 2. Materials and Methods

### 2.1. Molecular Modeling and Peptide Docking 

BioPredict Inc. proprietary software (https://www.biopredict.com December of 2022) and Discovery Studio software (Dassault Systems, BIOVIA Corp., San Diego, CA, USA, https://www.3ds.com/products/biovia/discovery-studio December of 2022) were used for molecular modeling and docking. For energy minimization and molecular dynamics simulations, Charmm (https://www.academiccharmm.org December of 2022) [33] and Gromacs packages (https://www.gromacs.org December of 2022) [34] were used. 

### 2.2. In Vitro Time-Resolved FRET Assay

Commercial PD-1/PD-L1 binding assay kit (CisBio, currently Perkin-Elmer, Bedford, MA) for time resolve fluorescent energy transfer (FRET) was used to assess peptides binding to PD-1. The assay for peptide screening was performed in a 96-well format (normal pressure), starting with 4-fold serial dilutions of each peptide in triplicate. No-peptide triplicate wells were used to assess fluorescent energy transfer in the absence of inhibition, and no-PD-1/PD-L1 triplicate wells were used to assess the background fluorescent energy transfer in the absence of PD-1/PD-L1 complexes. More detailed concentration dependences were obtained for selected peptides. Only freshly dissolved peptides were used in the assay. 

### 2.3. NMR Experiments

Uniformly ^15^N-labeled PD-1 was overexpressed in M9 medium, supplemented with ^15^NH_4_Cl as the sole nitrogen source, and purified as described elsewhere [8]. We ran chemical shift mapping (CSM) experiments for four peptides, B5.11, B3.15, B5.6, and B2.22. The last one was too hydrophobic and precipitated totally out of the solution. Therefore, we obtained CSM data for three peptides. CSM ^15^N-HSQC experiments with unlabeled peptides (B5.11, B3.15, and B5.6) were performed on uniformly ^15^N-labeled PD-1 in a buffer (two series, pH 6.4 and 6.7) containing 25 mM NaPO_4_, 100 mM NaCl, 1 mM DSS, 10% D2O, and 3% dDMSO (necessary to make peptides stocks). Spectra were recorded at 25 °C on a Varian INOVA 600 MHz spectrometer equipped with an inverse triple-resonance cold probe. Spectra were processed using NMRPipe (11.2 rev 2022.346.12.26) [35] and analyzed with CCPN Analysis [36] made available through NMRBox. Assignments for PD-1 were adopted from BMRB-18908. The chemical shift perturbations were calculated using the following equation [37]:

$$\Delta \delta =\sqrt{0.5({\delta^{2}}_{H}+({\alpha \delta_{N})}^{2}})$$

where the scaling factor, α, was set to 0.15.

We also performed transferred NOEs (trNOE) experiments for B5.11 peptide in a complex with unlabeled PD1. We tested several peptide-to-protein ratios within the 150:1 to 15:1 range: the optimal, with maximum additional peaks, being close to the lower limit. We used these data for the structural calculation of the B5.11 peptide ensemble as a part of its complex with PD-1.

Transferred NOEs for B5.11 peptide in complex with unlabeled PD1 were obtained at a peptide-to-protein ratio of 15:1, with a mixing time of 400 ms, in a buffer (pH 6.4) containing 25 mM NaPO_4_, 8.3 mM NaOAc, 11 mM NH_4_CO_3_, 1 mM DSS, and 10% D2O. The 1H assignments for the peptide were acquired through the combination of COSY, TOCSY, NOESY, and natural abundance ^15^N-HSQC. 

The B5.11 peptide ensemble (as a part of the complex with PD-1) was calculated based upon trNOE restraints using ARIA 2.3 [38], and the ensemble of 15 structures with minimal overall energy was refined in explicit water. During the course of the calculations, the quality of the molecular structures was assessed with ARIA/CNS built-in scripts and PROCHECK-NMR.20 [39].

## 3. Results

### 3.1. Modeling of Peptide PD-1 Inhibitors Based on Known PD-1/PD-L1(-L2) Interfaces

**Figure 1:**
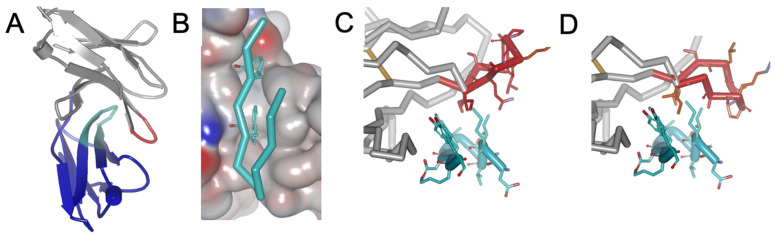
The X-ray structure of the complex (PDB id: 6umt), explored for presented modeling: (**A**) crisscross interaction between β-sheets of PD-1 (grey) and PD-L2 (blue); PD-L2 β-hairpin is shown in cyan and PD-1 FG loop in red; (**B**) surface charge map of PD-1 binding pocket with PD-L2 β-hairpin inside (in an orientation opposite to shown in panel (**A**); models: β-hairpin is shown in complex within an “open” (**C**) and a “closed” (**D**) conformation of PD-1 FG loop.

The ectodomain of PD-1 binds to ectodomains of PD-L1(-L2) by β-sheet-to-β-sheet crisscross interaction with the side of the PD-L1/2 β-sheet getting into the concaved part of PD-1 β-sheet (Figure 1A). Detailed information on specific interactions in these complexes was obtained from the X-ray structures determined for human and murine PD-1/PD-L1 and PD-1/PD-L2 dimers [9,21,23,40]. These structures identified two contiguous strands of PD-L1(-L2) β-sheet that form a critical β-hairpin, which binds to a specific interface in PD1 (Figure 1A). The binding interface, shown for PD-1 by the surface charge map (Figure 1B), indicates a pocket that encloses Y^112^ and W^110^ of PD-L2 β-hairpin one leg and a hydrophobic patch in contact with the other leg of this β-hairpin. Both induced fit and conformational selection might be envisioned from the available interfaces between human PD-1 and PD-L1/PD-L2, which were acquired through site-directed mutagenesis and X-ray crystallography by Tang et al. [23]: while the FG loop of PD-1 is predominantly “open” in an apo state (Figure 1C), it converts to, or accommodates, a predominantly “closed” conformation upon binding (Figure 1D). Critically, interactions between human PD-1 and PD-L1 and, particularly, PD-L2 induce significant conformational change in the FG loop of PD-1, resulting in an enhanced binding and stabilization of the complex.

### 3.2. Design and Validation of Peptide Inhibitors

Using the structures of the critical PD-L1/-L2 β-hairpins involved in binding to PD1, as a starting point, we designed several series of 12–14 amino acid peptides. The design followed the structural constraints of the target PD-1 protein footprint (ligand–protein interaction maps) derived from (i) numerous solved PD-1 structures with its cognate ligands and antibodies and (ii) sequence comparisons of PD-1, PD-L1, and PD-L2 from various species. 

Using natural and non-natural amino acids, two structurally different peptide folds were extensively explored. The 14-mer and a subset of 12-mer peptides were designed to form a β-hairpin-like structure. Another subset of 12-mer peptides was designed to have linear conformation, with eight C-terminal amino acids similar to one leg of β-hairpin and four N-terminal amino acids selected for binding to potential partners in the PD-1. 

**Figure 2:**
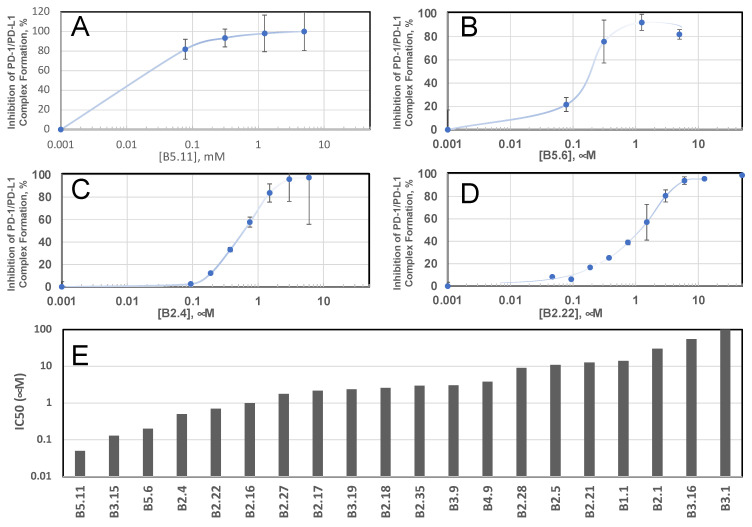
Ranking of the selected peptide inhibitors of PD-1/PD-L1 binding by in vitro TR FRET assay. B1.1 is the first tested peptide of the series. Panels (**A**,**B**) show examples of initial fourfold dilution data for peptides B5.11 and B5.6, respectively. Panels (**C**,**D**) present further detailed inhibition curves for peptides B2.4 and B2.22, respectively. STD values are plotted for the data points measured in triplicates. Representative IC_50_ values, covering three orders of magnitude range, are visualized in panel (**E**).

Various QSAR (quantitative structure activity relationship) hypotheses were tested by synthesizing batches of 10–12 peptides. In total, over one hundred candidates in ten iterations were screened for their ability to inhibit in vitro PD-1/PD-L1 binding using the commercial PD-1/PD-L1 binding assay kit (CisBio, currently Perkin-Elmer). Briefly, this kit uses ectodomains of PD-1 and PD-L1 that are expressed with orthogonal fusion tags (Tag-1 and Tag-2). It also includes two antibodies to selectively interact with these tags. The antibody against one tag is labeled with fluorescent moiety, which serves as a donor, while the other antibody is labeled with fluorescent moiety, which serves as an acceptor in fluorescent resonance energy transfer (FRET). When the complex is formed and probed with both anti-Tag-1 and anti-Tag-2 antibodies, donor and acceptor are sufficiently close, and FRET can be detected in a time-resolved (TR) manner. Inhibitors of PD-1/PD-L1 interactions prevent complex formation, leading to a decrease in TR FRET in a concentration-dependent manner, as shown in Figure 2 for four representative peptide inhibitors. Initial fourfold dilutions are exemplified by data for peptides B5.11 and B5.6 (panels A and B); examples of the more detailed follow-up concentration dependences are shown for peptides B2.4 and B2.22 (panels C and D). The potency of the inhibitors is then characterized by IC_50_, a concentration that inhibits TR FRET by 50%, allowing for a facile comparison of different inhibitors (Figure 2E). Sequences and IC_50_ values for the five most active peptides are presented in Table 1, together with the wild-type (WT) sequence of the corresponding region of PD-L2. A table with all peptides tested, including those that showed no inhibition, can be found in the Supplementary Materials (Supplementary Table S1).

**Figure 3:**
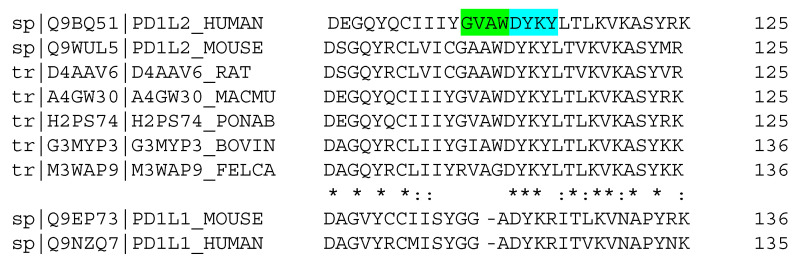
Sequence alignment of the selected region from mammalian PD-L2, in comparison to the one from PD-L1 of human and mouse origin (https://www.uniprot.org/align/clustalo-R20240507-004057-0210-34998008-p1m, accessed on 6 May 2024): “*“ sign marks identical residues in all compared sequences, “:” sign marks amino acids of the same type. Highly conserved residues discussed are highlighted in cyan (and green of for a less conserved).

Notably, both the 14- and 12-mer most active peptides shared eight C-terminal amino acid sequence GVAWDYKY, identical to one leg of a β-hairpin in WT PD-L2. Sequence alignments of PD-L1(-L2) from multiple species show a remarkable conservation of the WDYK motif (Figure 3). Consistent with this occurrence, all tested amino acid substitutions in this part of the peptide strongly decreased the peptide’s ability to inhibit PD1/PD-L1 complex formation. The N-terminus in the designed peptides can be rotated freely around the glycine in the middle (see Table 1). Therefore, the makeup of the N-terminal sequence determines the peptide fold. Our best peptide binders follow two folds: (i) a β-hairpin-like fold that mimics the fold of the wild-type PD-L2 hairpin and (ii) linear peptide series with four amino acids of N-terminal pointing out of the protein–protein interface cavity.

#### 3.2.1. β-Hairpin-like Fold

Following the PD-L2 β-hairpin interface with PD-1, we hypothesized that it is possible to design an optimized β-hairpin-like peptide that will force the flexible FG loop of PD-1 to make an *induced fit* that will lead to increased affinity. The first challenge in testing this hypothesis was to design a peptide that folds back on itself into a β-hairpin-like configuration. The second challenge was to design a β-hairpin that can change the conformation of the FG loop in PD-1 to enhance affinity. Our modeling shows the following binding dynamics for such a hypothetical peptide. Upon binding, one strand of our β-hairpin gets into the cavity of PD-1 (Figure 1B). Then, clustered hydrophobic residues of the other strand get under the hydrophobic patch of the protein–protein interaction site, stimulating the induced fit with the flexible loop, which now assumes the “closed” conformation (Figure 1D), thus stabilizing binding. Our modeling revealed that optimizing these hydrophobic interactions is an efficient path to high-affinity peptides. 

Indeed, the modeling indicates that N-terminal segments of peptides B5.6, B2.4, and B2.22 are folding back and positioning themselves under the FG loop in PD-1, inducing its conformational changes. The sequence of B2.22, a 12-amino-acid-long peptide, is very close to the “wild type” sequence, and we conjectured that it makes a β-hairpin similar to the corresponding part of the PD-L2 protein. Following B2.22, the N-termini of 14 amino acids long B5.6 and B2.4 also fold back and are positioned under the FG loop in PD-1. 

**Figure 4:**
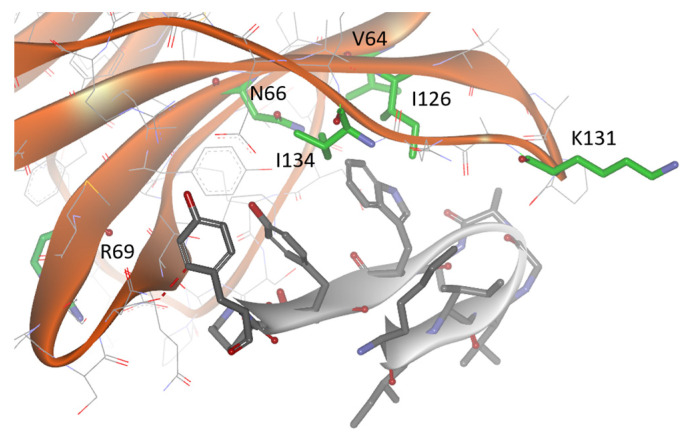
Model of the peptide B5.6 (colored in grey) in complex with PD-1 (PDB id: 2M2D, secondary structure in gold). Side chains of PD-1 residues near the interface that showed chemical shift perturbation in ^15^N-HSQC of peptide titration experiments are colored in green.

Chemical shifts mapping experiments, performed by the titration of B5.6 into ^15^N-labeled PD-1, supported the proposed model, as the residues V64, N66, R69, I126, I134, and K131 in the FG loop of PD-1 showed some chemical shift perturbations induced by peptide binding to PD-1 (see Figure 4). 

#### 3.2.2. Linear Peptides

**Figure 5:**
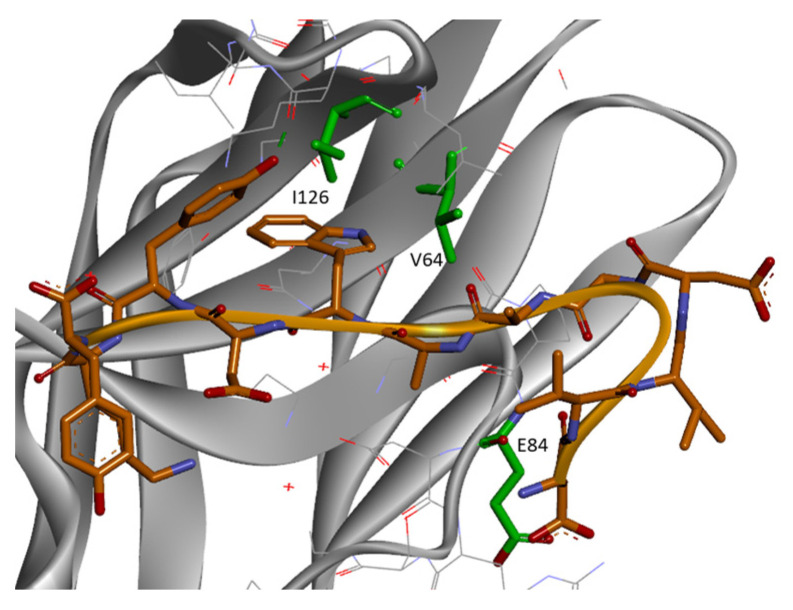
Model of the peptide B5.11 (colored in gold) in complex with PD-1 (PDB id: 5GGS, secondary structure in grey). Side chains of PD-1 residues near interface that showed chemical shift perturbation in ^15^N-HSQC of B3.15 titration are colored in green.

**Figure 6:**
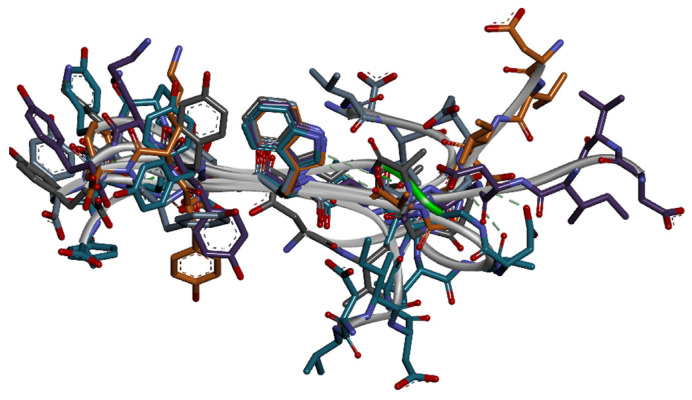
Ensemble of B5.11 conformations, bound to PD-1 state, is calculated based on distance restraints obtained from trNOE experiments.

The peptides assume stretched or “linear” conformation when a negative charge is on the N-terminus of the peptide–peptides B5.11 and B3.15 from Table 1. Because of the charge, the N-terminal cannot get under the hydrophobic FG-loop, and the FG-loop cannot fold over and induce conformational changes. Nevertheless, among linear peptides, we found two effective inhibitors of PD1/PD-L1 complex formation, B5.11 with IC_50_ of 0.05 µM and B3.15 with IC_50_ of 0.13 µM (Figure 5). Chemical shift mapping experiments, performed by the titration of B3.15 into ^15^N-labeled PD-1, confirmed that the binding of this peptide affects residues V64, I126, and E84, ascertaining that the C-terminus of B3.15 is in the cavity of PD-1. The trNOE NMR experiments, run on another peptide, B5.11, allowed for us to calculate the ensemble of B5.11 conformations in bound to PD-1. These conformations, aligned by the WDYK motif, showed good structural alignment of the linear C-terminal part of the peptide B5.11 (Figure 6, left side). The N-terminal region of the peptide, through the rotation over a glycine residue, exhibits fan-out conformations without making a peptide fold-over (Figure 6, right side). We docked this set of conformations to the PD-1 structure determined by NMR (PDB id: 5GGS) and selected the conformation that showed the best energy for the complex (Figure 5).

## 4. Discussion

**Figure 7:**
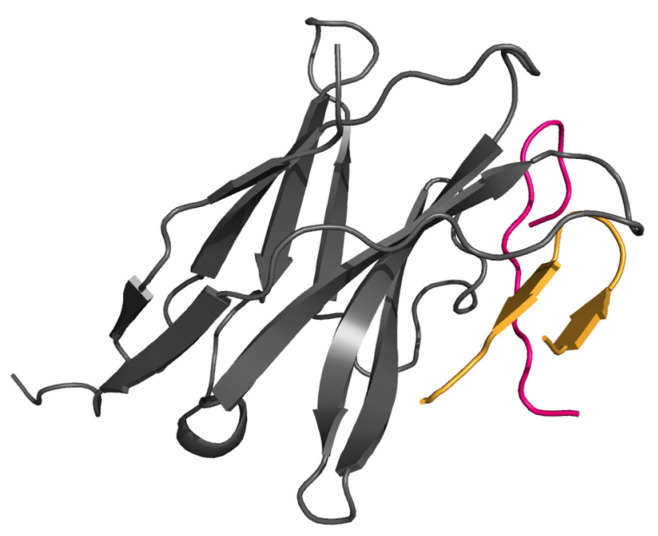
The overlay of the linear (pink) and β-hairpin (gold) peptides, alongside of PD-1, are presented to compare the placement.

Our most active peptides from both series, β-hairpin-like and linear, share an eight C-terminal amino acid sequence GVAWDYKY, identical to one leg a of β-hairpin in WT PD-L2, consistent with our original hypothesis. Interestingly, some of the linear peptides, in comparison to the hairpin-like, showed more favorable pharmacodynamic and pharmacokinetic properties. The former could be due to large entropic contributions, while the latter is because of much higher water solubility. According to our modeling, the representatives from two series interact with PD-1 in an overlapping but not totally identical mode (Figure 7), providing a wider conformational space for the design of potential peptidomimetic or macrocyclic peptide inhibitors. The FRET data for peptides with low or no inhibitory activity, provided in Supplementary Table S1, have their own value: they can be used for potential AI training in future.

## 5. Conclusions

Through an in-depth analysis of the available structural data reflecting the protein–protein interface, followed by an iterative process of binding testing, we developed a series of high-potency peptide inhibitors interfering with PD-1/PD-L1(-L2) interaction. Through NMR experiments, we confirmed some of our original reasoning, but also found somewhat unexpected conformations with favorable binding attributes. Our best peptide inhibitors are 12 and 14 amino acids long and show sub-micromolar IC_50_ inhibitory activity in the in vitro assay. The positioning of the peptides within the PD-1 binding site was established by extensive modeling supported by 2D NMR studies of PD-1/peptide complexes. Our results reflect substantial progress in the study of immune checkpoint inhibitors, providing a scaffold for the future development of novel peptidomimetics.

## Figures and Tables

**Table 1:** Sub-micromolar peptide inhibitors of PD-1/PD-L1 (as determined by TR FRET assay, IC_50_ shown in second row). C-terminal-conserved part of the peptide series is shown with a grey background. Charged aspartic acids of N-terminal are shown in red. Ac—acetated N-terminal; NH_2_—amidated C-terminal; NL—non-standard amino acid L-Norleucine.

PDL2	[μM]		Q	C	I	I	I	Y	G	V	A	W	D	Y	K	Y	
B5.11	0.05			Ac	D	V	I	D	G	A	A	W	D	Y	K	Y	NH_2_
B3.15	0.13			Ac	D	V	I	V	G	A	A	W	D	Y	K	Y	NH_2_
B5.6	0.2	Ac	NL	V	L	V	I	V	G	A	A	W	D	Y	K	Y	NH_2_
B2.4	0.5	Ac	R	V	NL	V	I	V	G	A	A	W	D	Y	K	Y	NH_2_
B2.22	0.7			Ac	L	V	I	V	G	A	A	W	D	Y	K	Y	NH_2_

## Data Availability

All NMR software used is available freely from NMRBox server: https://nmrbox.nmrhub.org. All raw NMR spectra collected are available from the corresponding author upon request. NMR spectra examples are provided in the Supporting Information .pdf file. Restraints derived from trNOE data used for B5.11 peptide ensemble calculation are provided in the Supporting Information .pdf file. Peptide B5.6 in complex with PD-1 (PDB id: 2M2D) is provided in the Supporting Information .pdb file. Peptide B5.11 in complex with PD-1 (PDB id: 5GGS) is provided in the Supporting Information .pdb file. The calculated ensemble of B5.11 peptide is provided in the Supporting Information .pdb file.

## Supplementary Materials

The following supporting information can be downloaded at: https://www.mdpi.com/article/10.3390/biom14050597/s1, PDF file containing examples of NMR spectra (Supplementary Figure S1), distance restraints (Supplementary Figure S2), and Supplementary Table S1 with all peptides tested: Supplementary Figure S1: (A) ^15^N-HSQC spectra: ^15^N labeled PD-1 (apo shown in red) was titrated with none-labeled peptide B5.11 (1:2 ratio shown in maroon, 1:4 in mauve, 1:8 in blue) and overlayed with natural abundance spectrum of B5.11 shown in teal; (B) Extended region of the HSQC spectra focusing on the amide peak of Val64 (apo in red) overlayed with peptide B5.6 titration at 1:10 ratio (shown in green); (C) Extended region of the HSQC spectra focusing on the amide peak of Val64 (apo in red) overlayed with peptide B5.11 titration (colors are the same as in panel A); (D) ^15^N-HSQC (natural abundance) spectrum of peptide B5.11 is presented along with the assignments; E) Overlay of peptide B5.11 TOCSY spectrum (shown in blue, along with the assignments) with NOESY (shown in black) and trNOESY (shown in magenta) spectra. Supplementary Figure S2: Distance restraints, derived from trNOE data, which were used for B5.11 peptide ensemble calculations are listed. Supplementary Table S1: All peptides, tested to inhibit PD-1/PD-L1 binding by in vitro TR-FRET assay, and corresponding IC_50_ are presented. “NI“ sign is posted when no inhibition was found with maximum concentration (*—5 or **—50 μM) tested. PDB file containing peptide B5.6 in complex with PD-1; peptide B5.11 in complex with PD-1; and ensemble of B5.11 peptide conformations (in bound to PD-1 state).
