# Supplementary material for: In Search of Better Peptide-(Derived from PD-L2)-Based Immune Checkpoint Inhibitors
Source: Biomolecules. 2024 May 18;14(5):597. doi: 10.3390/biom14050597 (PMC11118832; doi:10.3390/biom14050597)
Supplement: Supplementary file 1 [file biomolecules-14-00597-s001.zip › biomolecules-2984796-Supplementary.pdf]

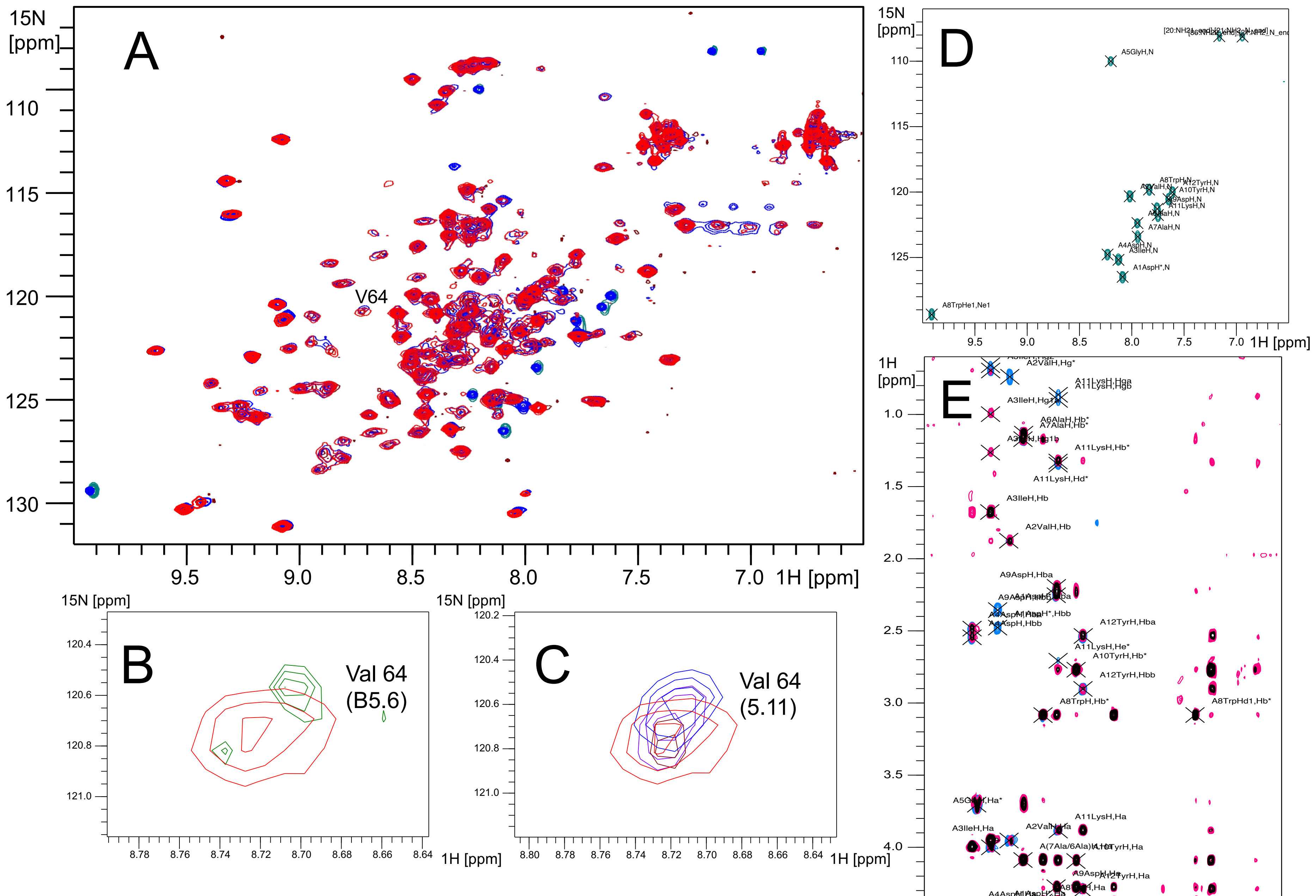

Supplementary Figure S1

Supplementary Figure S2

```
assign (segid "   A" and resid    3 and name   HN) (segid "   A" and
resid    3 and name HG12) 3.504 1.534 1.534 weight 1.000 !
spec=VJNO...oPep, no=3, id=2, vol=1.033169e-02
assign (segid "   A" and resid    6 and name   HN) (segid "   A" and
resid    6 and name  HB2) 2.507 0.786 0.786 weight 1.000 !
spec=VJNO...oPep, no=6, id=5, vol=6.939026e-02
    or (segid "   A" and resid    6 and name   HN) (segid "   A" and
resid    6 and name  HB1)
    or (segid "   A" and resid    6 and name   HN) (segid "   A" and
resid    6 and name  HB3)
assign (segid "   A" and resid    3 and name   HN) (segid "   A" and
resid    3 and name HG11) 3.373 1.423 1.423 weight 1.000 !
spec=VJNO...oPep, no=7, id=6, vol=1.320012e-02
assign (segid "   A" and resid    3 and name   HN) (segid "   A" and
resid    3 and name   HB) 2.775 0.962 0.962 weight 1.000 !
spec=VJNO...oPep, no=9, id=8, vol=4.278010e-02
assign (segid "   A" and resid    2 and name   HN) (segid "   A" and
resid    2 and name   HB) 3.049 1.162 1.162 weight 1.000 !
spec=VJNO...oPep, no=10, id=9, vol=2.340396e-02
assign (segid "   A" and resid   12 and name   HN) (segid "   A" and
resid   12 and name  HB1) 3.407 1.451 1.451 weight 1.000 !
spec=VJNO...oPep, no=14, id=13, vol=1.229326e-02
assign (segid "   A" and resid   10 and name   HN) (segid "   A" and
resid   10 and name  HB1) 2.245 0.630 0.630 weight 1.000 !
spec=VJNO...oPep, no=16, id=15, vol=1.315039e-01
assign (segid "   A" and resid   12 and name   HN) (segid "   A" and
resid   12 and name  HB2) 2.950 1.088 1.088 weight 1.000 !
spec=VJNO...oPep, no=17, id=16, vol=2.831155e-02
assign (segid "   A" and resid   12 and name  HD1) (segid "   A" and
resid   12 and name  HB2) 2.696 0.909 0.909 weight 1.000 !
spec=VJNO...oPep, no=22, id=21, vol=4.948945e-02
    or (segid "   A" and resid   12 and name  HD2) (segid "   A" and
resid   12 and name  HB2)
assign (segid "   A" and resid   12 and name  HD1) (segid "   A" and
resid   12 and name  HB1) 2.707 0.916 0.916 weight 1.000 !
spec=VJNO...oPep, no=26, id=25, vol=4.610408e-02
    or (segid "   A" and resid   12 and name  HD2) (segid "   A" and
resid   12 and name  HB1)
assign (segid "   A" and resid    5 and name   HN) (segid "   A" and
resid    4 and name   HA) 2.909 1.058 1.058 weight 1.000 !
spec=VJNO...oPep, no=29, id=27, vol=2.967369e-02
assign (segid "   A" and resid    2 and name   HN) (segid "   A" and
resid    1 and name   HA) 2.664 0.887 0.887 weight 1.000 !
spec=VJNO...oPep, no=30, id=28, vol=5.278222e-02
assign (segid "   A" and resid    4 and name   HN) (segid "   A" and
resid    3 and name   HA) 2.391 0.715 0.715 weight 1.000 !
spec=VJNO...oPep, no=32, id=30, vol=9.453267e-02
assign (segid "   A" and resid    2 and name   HN) (segid "   A" and
```

```
resid    2 and name   HA) 3.107 1.207 1.207 weight 1.000 !
spec=VJNO...oPep, no=33, id=31, vol=1.611717e-02
assign (segid "   A" and resid    3 and name   HN) (segid "   A" and
resid    2 and name   HA) 2.221 0.616 0.616 weight 1.000 !
spec=VJNO...oPep, no=34, id=32, vol=1.572275e-01
assign (segid "   A" and resid    8 and name   HN) (segid "   A" and
resid    7 and name   HA) 2.389 0.714 0.714 weight 1.000 !
spec=VJNO...oPep, no=38, id=36, vol=9.253265e-02
assign (segid "   A" and resid   10 and name   HN) (segid "   A" and
resid   10 and name   HA) 2.574 0.828 0.828 weight 1.000 !
spec=VJNO...oPep, no=40, id=38, vol=5.693072e-02
assign (segid "   A" and resid   11 and name   HN) (segid "   A" and
resid   11 and name   HA) 3.060 1.171 1.171 weight 1.000 !
spec=VJNO...oPep, no=41, id=39, vol=2.088676e-02
assign (segid "   A" and resid   12 and name   HN) (segid "   A" and
resid   11 and name   HA) 2.736 0.935 0.935 weight 1.000 !
spec=VJNO...oPep, no=42, id=40, vol=4.465870e-02
assign (segid "   A" and resid    8 and name   HN) (segid "   A" and
resid    8 and name   HA) 2.836 1.006 1.006 weight 1.000 !
spec=VJNO...oPep, no=43, id=41, vol=3.252475e-02
assign (segid "   A" and resid    9 and name   HN) (segid "   A" and
resid    8 and name   HA) 2.527 0.798 0.798 weight 1.000 !
spec=VJNO...oPep, no=44, id=42, vol=7.001019e-02
assign (segid "   A" and resid   12 and name   HN) (segid "   A" and
resid   12 and name   HA) 3.068 1.177 1.177 weight 1.000 !
spec=VJNO...oPep, no=46, id=44, vol=2.023048e-02
assign (segid "   A" and resid    9 and name   HN) (segid "   A" and
resid    9 and name   HA) 2.762 0.953 0.953 weight 1.000 !
spec=VJNO...oPep, no=47, id=45, vol=3.840271e-02
assign (segid "   A" and resid   10 and name   HN) (segid "   A" and
resid    9 and name   HA) 2.389 0.714 0.714 weight 1.000 !
spec=VJNO...oPep, no=48, id=46, vol=9.143525e-02
assign (segid "   A" and resid    8 and name  HE3) (segid "   A" and
resid    9 and name   HA) 3.141 1.233 1.233 weight 1.000 !
spec=VJNO...oPep, no=49, id=47, vol=1.930137e-02
assign (segid "   A" and resid   10 and name  HD1) (segid "   A" and
resid   10 and name   HA) 2.507 0.786 0.786 weight 1.000 !
spec=VJNO...oPep, no=53, id=51, vol=7.056875e-02
    or (segid "   A" and resid   10 and name  HD2) (segid "   A" and
resid   10 and name   HA)
assign (segid "   A" and resid   12 and name  HD1) (segid "   A" and
resid   12 and name   HA) 2.680 0.898 0.898 weight 1.000 !
spec=VJNO...oPep, no=54, id=52, vol=4.880517e-02
    or (segid "   A" and resid   12 and name  HD2) (segid "   A" and
resid   12 and name   HA)
assign (segid "   A" and resid    8 and name   HN) (segid "   A" and
resid    8 and name  HD1) 3.304 1.364 1.364 weight 1.000 !
spec=VJNO...oPep, no=55, id=53, vol=1.313896e-02
assign (segid "   A" and resid   10 and name   HN) (segid "   A" and
resid   10 and name  HD1) 3.024 1.143 1.143 weight 1.000 !
```

```
spec=VJNO...oPep, no=56, id=54, vol=2.097030e-02
    or (segid "   A" and resid   10 and name   HN) (segid "   A" and
resid   10 and name  HD2)
assign (segid "   A" and resid    9 and name   HN) (segid "   A" and
resid    8 and name  HE3) 3.722 1.731 1.731 weight 1.000 !
spec=VJNO...oPep, no=69, id=63, vol=6.442704e-03
assign (segid "   A" and resid   10 and name   HN) (segid "   A" and
resid    9 and name   HN) 3.412 1.455 1.455 weight 1.000 !
spec=VJNO...oPep, no=77, id=68, vol=1.185256e-02
assign (segid "   A" and resid    9 and name   HN) (segid "   A" and
resid    8 and name   HN) 3.524 1.552 1.552 weight 1.000 !
spec=VJNO...oPep, no=78, id=69, vol=8.638570e-03
assign (segid "   A" and resid    2 and name   HN) (segid "   A" and
resid    3 and name   HN) 3.378 1.426 1.426 weight 1.000 !
spec=VJNO...oPep, no=82, id=73, vol=1.166580e-02
assign (segid "   A" and resid    4 and name   HN) (segid "   A" and
resid    3 and name   HN) 3.581 1.603 1.603 weight 1.000 !
spec=VJNO...oPep, no=84, id=75, vol=8.476181e-03
assign (segid "   A" and resid   10 and name  HD1) (segid "   A" and
resid    8 and name   HA) 3.526 1.554 1.554 weight 1.000 !
spec=VJNO...oPep, no=96, id=76, vol=9.068958e-03
    or (segid "   A" and resid   10 and name  HD2) (segid "   A" and
resid    8 and name   HA)
assign (segid "   A" and resid    3 and name   HN) (segid "   A" and
resid    2 and name   HB) 3.673 1.686 1.686 weight 1.000 !
spec=VJNO...oPep, no=100, id=78, vol=7.676500e-03
assign (segid "   A" and resid    4 and name   HN) (segid "   A" and
resid    3 and name HG22) 3.783 1.789 1.789 weight 1.000 !
spec=VJNO...oPep, no=103, id=81, vol=5.483137e-03
    or (segid "   A" and resid    4 and name   HN) (segid "   A" and
resid    3 and name HG21)
    or (segid "   A" and resid    4 and name   HN) (segid "   A" and
resid    3 and name HG23)
assign (segid "   A" and resid   12 and name  HE1) (segid "   A" and
resid   11 and name  HG1) 3.722 1.732 1.732 weight 1.000 !
spec=VJNO...oPep, no=105, id=82, vol=6.300575e-03
    or (segid "   A" and resid   12 and name  HE2) (segid "   A" and
resid   11 and name  HG1)
assign (segid "   A" and resid    8 and name  HD1) (segid "   A" and
resid    8 and name  HE1) 2.166 0.587 0.587 weight 1.000 !
spec=VJNO...oPep, no=113, id=88, vol=1.429870e-01
assign (segid "   A" and resid    8 and name  HE1) (segid "   A" and
resid    7 and name   HA) 3.776 1.782 1.782 weight 1.000 !
spec=VJNO...oPep, no=114, id=89, vol=5.481763e-03
assign (segid "   A" and resid    7 and name   HN) (segid "   A" and
resid    7 and name  HB2) 2.715 0.921 0.921 weight 1.000 !
spec=VJNO...oPep, no=116, id=91, vol=4.317954e-02
    or (segid "   A" and resid    7 and name   HN) (segid "   A" and
resid    7 and name  HB1)
    or (segid "   A" and resid    7 and name   HN) (segid "   A" and
```

```
resid    7 and name  HB3)
assign (segid "   A" and resid    3 and name   HA) (segid "   A" and
resid    3 and name HG22) 2.700 0.911 0.911 weight 1.000 !
spec=VJNO...oPep, no=118, id=92, vol=4.247479e-02
    or (segid "   A" and resid    3 and name   HA) (segid "   A" and
resid    3 and name HG21)
    or (segid "   A" and resid    3 and name   HA) (segid "   A" and
resid    3 and name HG23)
assign (segid "   A" and resid    3 and name   HA) (segid "   A" and
resid    3 and name HG11) 3.165 1.252 1.252 weight 1.000 !
spec=VJNO...oPep, no=119, id=93, vol=1.759148e-02
assign (segid "   A" and resid    7 and name   HA) (segid "   A" and
resid    7 and name  HB2) 2.138 0.572 0.572 weight 1.000 !
spec=VJNO...oPep, no=120, id=94, vol=1.905652e-01
    or (segid "   A" and resid    7 and name   HA) (segid "   A" and
resid    7 and name  HB1)
    or (segid "   A" and resid    7 and name   HA) (segid "   A" and
resid    7 and name  HB3)
assign (segid "   A" and resid    6 and name   HA) (segid "   A" and
resid    6 and name  HB2) 2.176 0.592 0.592 weight 1.000 !
spec=VJNO...oPep, no=121, id=95, vol=1.708650e-01
    or (segid "   A" and resid    6 and name   HA) (segid "   A" and
resid    6 and name  HB1)
    or (segid "   A" and resid    6 and name   HA) (segid "   A" and
resid    6 and name  HB3)
assign (segid "   A" and resid    3 and name   HA) (segid "   A" and
resid    3 and name   HB) 2.760 0.952 0.952 weight 1.000 !
spec=VJNO...oPep, no=125, id=99, vol=3.883141e-02
assign (segid "   A" and resid    2 and name   HA) (segid "   A" and
resid    2 and name   HB) 3.034 1.151 1.151 weight 1.000 !
spec=VJNO...oPep, no=131, id=100, vol=2.151193e-02
assign (segid "   A" and resid   12 and name   HA) (segid "   A" and
resid   12 and name  HB1) 2.585 0.835 0.835 weight 1.000 !
spec=VJNO...oPep, no=133, id=102, vol=6.225432e-02
assign (segid "   A" and resid   12 and name   HA) (segid "   A" and
resid   12 and name  HB2) 2.883 1.039 1.039 weight 1.000 !
spec=VJNO...oPep, no=134, id=103, vol=3.120618e-02
assign (segid "   A" and resid    8 and name  HD1) (segid "   A" and
resid    7 and name   HA) 3.085 1.190 1.190 weight 1.000 !
spec=VJNO...oPep, no=155, id=123, vol=2.121782e-02
assign (segid "   A" and resid   12 and name   HA) (segid "   A" and
resid   12 and name  HE1) 3.590 1.611 1.611 weight 1.000 !
spec=VJNO...oPep, no=157, id=125, vol=8.239377e-03
    or (segid "   A" and resid   12 and name   HA) (segid "   A" and
resid   12 and name  HE2)
assign (segid "   A" and resid    8 and name   HA) (segid "   A" and
resid   10 and name  HE1) 3.636 1.653 1.653 weight 1.000 !
spec=VJNO...oPep, no=167, id=135, vol=7.649898e-03
    or (segid "   A" and resid    8 and name   HA) (segid "   A" and
resid   10 and name  HE2)
```

```
assign (segid "   A" and resid    3 and name   HB) (segid "   A" and
resid    3 and name HG11) 3.006 1.130 1.130 weight 1.000 !
spec=VJNO...oPep, no=169, id=137, vol=2.488300e-02
assign (segid "   A" and resid    3 and name   HB) (segid "   A" and
resid    3 and name HG12) 2.929 1.072 1.072 weight 1.000 !
spec=VJNO...oPep, no=170, id=138, vol=3.032798e-02
assign (segid "   A" and resid    3 and name HG11) (segid "   A" and
resid    3 and name HG12) 2.185 0.597 0.597 weight 1.000 !
spec=VJNO...oPep, no=173, id=141, vol=1.668313e-01
assign (segid "   A" and resid    7 and name  HB2) (segid "   A" and
resid    8 and name   HA) 3.507 1.537 1.537 weight 1.000 !
spec=VJNO...oPep, no=182, id=147, vol=9.864725e-03
    or (segid "   A" and resid    7 and name  HB1) (segid "   A" and
resid    8 and name   HA)
    or (segid "   A" and resid    7 and name  HB3) (segid "   A" and
resid    8 and name   HA)
assign (segid "   A" and resid    9 and name   HN) (segid "   A" and
resid    9 and name  HB1) 3.456 1.493 1.493 weight 1.000 !
spec=VJNO...oPep, no=192, id=154, vol=1.021430e-02
assign (segid "   A" and resid    9 and name   HN) (segid "   A" and
resid    9 and name  HB2) 3.535 1.562 1.562 weight 1.000 !
spec=VJNO...oPep, no=193, id=155, vol=9.293246e-03
assign (segid "   A" and resid   12 and name   HN) (segid "   A" and
resid   12 and name  HD1) 3.682 1.695 1.695 weight 1.000 !
spec=VJNO...oPep, no=200, id=159, vol=6.752008e-03
    or (segid "   A" and resid   12 and name   HN) (segid "   A" and
resid   12 and name  HD2)
assign (segid "   A" and resid   12 and name  HD1) (segid "   A" and
resid   11 and name  HG1) 3.936 1.936 1.936 weight 1.000 !
spec=VJNO...oPep, no=205, id=163, vol=4.989948e-03
    or (segid "   A" and resid   12 and name  HD2) (segid "   A" and
resid   11 and name  HG1)
assign (segid "   A" and resid   12 and name  HE1) (segid "   A" and
resid   12 and name  HB2) 3.819 1.823 1.823 weight 1.000 !
spec=VJNO...oPep, no=210, id=166, vol=5.159995e-03
    or (segid "   A" and resid   12 and name  HE2) (segid "   A" and
resid   12 and name  HB2)
assign (segid "   A" and resid    8 and name  HD1) (segid "   A" and
resid    7 and name  HB2) 3.613 1.631 1.631 weight 1.000 !
spec=VJNO...oPep, no=215, id=170, vol=6.971925e-03
    or (segid "   A" and resid    8 and name  HD1) (segid "   A" and
resid    7 and name  HB1)
    or (segid "   A" and resid    8 and name  HD1) (segid "   A" and
resid    7 and name  HB3)
assign (segid "   A" and resid    3 and name   HB) (segid "   A" and
resid    3 and name HG22) 2.251 0.634 0.634 weight 1.000 !
spec=VJNO...oPep, no=225, id=179, vol=1.082322e-01
    or (segid "   A" and resid    3 and name   HB) (segid "   A" and
resid    3 and name HG21)
    or (segid "   A" and resid    3 and name   HB) (segid "   A" and
```

```
resid    3 and name HG23)
assign (segid "   A" and resid    3 and name HG12) (segid "   A" and
resid    3 and name HD12) 2.383 0.710 0.710 weight 1.000 !
spec=VJNO...oPep, no=226, id=180, vol=9.003468e-02
    or (segid "   A" and resid    3 and name HG12) (segid "   A" and
resid    3 and name HD11)
    or (segid "   A" and resid    3 and name HG12) (segid "   A" and
resid    3 and name HD13)
assign (segid "   A" and resid    3 and name HG11) (segid "   A" and
resid    3 and name HD12) 2.260 0.638 0.638 weight 1.000 !
spec=VJNO...oPep, no=227, id=181, vol=1.220956e-01
    or (segid "   A" and resid    3 and name HG11) (segid "   A" and
resid    3 and name HD11)
    or (segid "   A" and resid    3 and name HG11) (segid "   A" and
resid    3 and name HD13)
assign (segid "   A" and resid   11 and name  HG1) (segid "   A" and
resid   11 and name   HA) 3.166 1.253 1.253 weight 1.000 !
spec=VJNO...oPep, no=235, id=186, vol=1.643317e-02
assign (segid "   A" and resid    3 and name   HA) (segid "   A" and
resid    3 and name   HN) 3.061 1.171 1.171 weight 1.000 !
spec=VJNO...oPep, no=237, id=187, vol=2.335292e-02
```

Supplementary Table S1

name	EC50 [µM]	peptide sequence																	
B5.11	0.05			Ac	D	V	NI*	D	G	A	A	W	D	Y	K	Y	NH2		
B3.15	0.13			Ac	D	V	NI*	V	G	A	A	W	D	Y	K	Y	NH2		
B5.6	0.2	Ac	NorLeu	V	L	V	NI*	V	G	A	A	W	D	Y	K	Y	NH2		
B2.4	0.5	Ac	R	V	NorLeu	V	NI*	V	G	A	A	W	D	Y	K	Y	NH2		
B2.22	0.7			Ac	L	V	NI*	V	G	A	A	W	D	Y	K	Y	NH2		
B2.16	1.0	Ac	NorLeu	V	L	V	NI*	V	G	A	A	W	D	R	K	Y	NH2		
B2.18	1.0	Ac	R	V	L	V	W	V	G	A	A	W	D	Y	K	Y	NH2		
B2.27	1.8	Ac	R	V	HomoLeu	V	HomoLeu	V	G	A	A	W	D	Y	K	Y	NH2		
B8.1	2.2	Ac	D	V	NI*	E	G	A	A	W	D	Y	K	Y	NH2				
B2.17	4	Ac	R	V	L	V	NorLeu	V	G	A	A	W	D	Y	K	Y	NH2		
B3.19	5			Ac	L	V	W	V	G	A	A	W	D	R	K	Y	NH2		
B4.9	5	Ac	R	V	NorLeu	V	NI*	V	G	A	Orn	W	D	Y	K	Y	NH2		
B8.11	5.8	Ac	D	V	NI*	D	G	P	A	W	D	Y	K	Y	NH2				
B8.7	6.8	Ac	D	V	NI*	D	G	A	A	W	D	Y	R	Y	NH2				
B3.9	8.5			Ac	L	V	NI*	V	G	P	A	W	D	Y	K	Y	NH2		
B2.35	10	K	R	V	L	V	NI*	V	G	A	A	W	D	Y	K	Y	E		
B2.5	11	Ac	NorLeu	V	L	V	NI*	V	G	A	A	W	D	Y	K	R	NH2		
B2.28	11		R	V	L	V	NI*	V	G	A	A	W	D	Y	K	Y			
B2.21	12.7	Ac	R	V	F	V	F	V	G	A	A	W	D	Y	K	Y	NH2		
B3.11	14				L	V	NI*	V	G	A	A	W	D	Y	K	Y			
B2.12	20	Ac	R	V	W	V	NI*	V	G	A	A	W	D	Y	K	Y	NH2		
B3.4	21			Ac	L	V	NI*	V	G	A	A	W	D	Y	K	R	NH2		
B3.13	21			Ac	L	V	NI*	C	G	A	C	W	D	Y	K	Y	NH2		
B2.26	22	Ac	Y	V	L	V	NI*	V	G	A	A	W	D	K	K	Y	NH2		
B2.32	22	Ac	R	V	L	V	NI*	V	G	A	A	HomoLeu	D	Y	K	Y	NH2		
B1.1	24	Ac	R	S	M	NI*	S	Y	G	G	A	D	D	Y	K	NH2			
B3.7	26			Ac	L	V	NI*	V	G	A	R	W	D	Y	K	R	NH2		
B2.1	30	Ac	R	V	L	V	NI*	V	G	A	A	W	D	Y	K	Y	NH2		
PL-120	35	Ac	G	A	D	Y	K	R	NI*	T	V	K	V	N	NH2				
B3.3	35			Ac	HomoLeu	V	HomoLeu	V	G	A	A	W	D	Y	K	Y	NH2		
CRVL-Cyclic	35		C	R	V	L	V	NI*	V	G	A	A	W	D	Y	K	Y		
B2.30	35	Ac	R	V	L	V	NI*	V	G	A	A	L	D	Y	K	Y	NH2		
B2.7	55	Ac	R	V	L	V	NI*	V	G	A	A	W	D	Y	K	R	NH2		
B2.11	55	Ac	R	V	K	V	NI*	V	G	A	A	W	D	Y	K	Y	NH2		
B3.20	NI*			Ac	HomoLeu	V	HomoLeu	V	G	A	A	W	D	R	K	Y	NH2		
B3.21	NI*			Ac	HomoLeu	V	W	V	G	A	A	W	D	R	K	Y	NH2		
B3.22	NI*			Ac	L	V	NI*	V	G	A	Orn	W	D	Y	K	Y	NH2		
B3.23	NI*			Ac	HomoLeu	V	W	V	G	A	Orn	W	D	R	K	Y	NH2		
B4.1	NI*			Ac	NorLeu	V	NI*	V	G	A	A	W	D	Y	K	Y	NH2		
B4.2	NI*				NorLeu	V	NI*	V	G	A	A	W	D	Y	K	Y			
B4.3	NI*			Ac	NorLeu	V	NI*	V	G	A	A	W	D	R	K	Y	NH2		
B4.4	NI*		R	V	NorLeu	V	W	V	G	A	A	W	D	Y	K	Y			
B4.5	NI*		R	V	NorLeu	V	NI*	V	G	A	A	W	D	Y	K				
B4.6	NI*	Ac	R	V	NorLeu	R	NI*	E	G	A	A	W	D	Y	K	Y	NH2		
B4.7	NI*		R	V	NorLeu	V	NI*	V	G	A	A	W	D	Y	K	Y			
B4.8	NI*		R	V	NorLeu	V	NorLeu	V	G	A	A	W	D	Y	K	Y			
B4.10	NI*		R	V	NorLeu	V	NI*	V	G	A	A	W	D	L-4-NH2-Phe	K	Y			
B4.11	NI*			Ac	NorLeu	V	W	V	G	A	A	W	D	Y	K	Y	NH2		
B4.12	NI*			Ac	NorLeu	V	W	V	G	A	A	W	D	R	K	Y	NH2		
B4.13	NI*				NorLeu	V	W	V	G	A	A	W	D	R	K	Y			
B4.14	NI*				NorLeu	V	NI*	V	G	A	A	W	D	Y	K	R			
B4.15	NI*				NorLeu	V	W	V	G	A	A	W	D	Y	K	Y			
B5.2	NI*			Ac	D	V	W	V	G	A	A	W	D	Y	K	Y	NH2		
B5.3	NI*			Ac	D	Homo Leu	NI*	V	G	A	A	W	D	Y	K	Y	NH2		
B5.5	NI*	Ac	NorLeu	V	D	V	NI*	V	G	A	A	W	D	Y	K	Y	NH2		
B5.7	NI*			Ac	D	NorLeu	NI*	V	G	A	A	W	D	Y	K	Y	NH2		
B5.8	NI*			Ac	D	V	NorLeu	V	G	A	A	W	D	Y	K	Y	NH2		
B5.10	NI*			Ac	D	NI*	NI*	V	G	A	A	W	D	Y	K	Y	NH2		
B5.12	NI*			Ac	E	V	NI*	V	G	A	A	W	D	Y	K	Y	NH2		
B5.13	NI*			Ac	D	W	NI*	V	G	A	A	W	D	Y	K	Y	NH2		
B5.14	NI*				D	V	NI*	V	G	A	A	W	D	Y	K	Y			
B6.1	NI*			Ac	D	V	NI*	V	G	A	A	L-1-Nal	D	Y	K	Y	NH2		
B6.2	NI*			Ac	L	V	NI*	V	G	A	A	W	pSer	Y	K	Y	NH2		
B6.3	NI*			Ac	D	V	NI*	V	G	A	A	W	D	Lys(Me1)	K	Y	NH2		
B7.1	NI*			Ac	D	V	NI*	V	G	A	A	W	D	Y	K	Y	L	T	NH2
B7.2	NI*			Ac	D	V	NI*	V	G	S	A	W	D	Y	K	Y	NH2		
B7.3	NI*			Ac	D	V	NI*	V	G	A	S	W	D	Y	K	Y	NH2		
B7.4	NI*			Ac	D	V	NI*	V	G	A	A	W	D	Y	K	Y	L	Y	NH2
B7.5	NI*			Ac	D	V	NI*	V	G	A	A	W	D	Y	K	Y	L	Q	NH2
B7.6	NI*			Ac	D	D	NI*	V	G	A	A	W	D	Y	K	Y	NH2		
B7.7	NI*			Ac	N	V	NI*	V	G	A	A	W	D	Y	K	Y	NH2		
B7.8	NI*			Ac	D	V	NI*	D	G	A	A	W	D	Y	K	Y	L	Q	NH2
B7.9	NI*			Ac	D	V	NI*	D	G	A	A	W	D	Y	K	Y	L	T	NH2
B7.10	NI*			Ac	D	V	NI*	Y	G	A	A	W	D	Y	K	Y	NH2		

B7.11	NI*	Ac	D	V	NI*	Y	G	A	A	W	D	Y	K	Y	L	T	NH2
B8.2	NI*	Ac	D	A	NI*	D	G	A	A	W	D	Y	K	Y	NH2		
B8.3	NI*	Ac	D	V	V	D	G	A	A	W	D	Y	K	Y	NH2		
B8.4	NI*	Ac	D	V	NI*	N	G	A	A	W	D	Y	K	Y	NH2		
B8.5	NI*	Ac	N	V	NI*	D	G	A	A	W	D	Y	K	Y	NH2		
B8.12	NI*	Ac	D	V	NI*	D	P	A	A	W	D	Y	K	Y	NH2		
B8.13	NI*	Ac	D	V	L	D	G	A	A	W	D	Y	K	Y	NH2		
B8.14	NI*	Ac	D	V	Homoleu	D	G	A	A	W	D	Y	K	Y	NH2		
B8.15	NI*	Ac	D	L	NI*	D	G	A	A	W	D	Y	K	Y	NH2		
B8.16	NI*	Ac	D	NI*	NI*	D	G	A	A	W	D	Y	K	Y	NH2		
B8.17	NI*	Ac	E	V	NI*	D	G	A	A	W	D	Y	K	Y	NH2		
B8.18	NI*	Ac	D	Homoleu	NI*	D	G	A	A	W	D	Y	K	Y	NH2		
B8.19	NI*	Ac	D(D)	V	NI*	D	G	A	A	W	D	Y	K	Y	NH2		
B8.20	NI*	Ac	D	V	NI*	D	G	A	D	W	D	Y	K	Y	NH2		
B8.21	NI*	Ac	D	V	NI*	D	G	A	A	H	D	Y	K	Y	NH2		
B8.22	NI*	Ac	D	V	NI*	D	G	A	A	W	D	R	K	Y	NH2		
B8.23	NI*	Ac	D	V	NI*	D	G	A	A	W	D	Y	K	R	NH2		
B8.24	NI*	Ac	D	V	NI*	D	G	A	A	W	D	Y	K	NH2			
B9.0	NI*				Ac	D	G	A	A	W	D	Y	K	Y	NH2		
B9.1	NI*					Ac	D	A	A	W	D	Y	K	Y	NH2		
B9.2	NI*						Ac	D	A	W	D	Y	K	Y	NH2		
B9.3	NI*						Ac	D(D)	A	W	D	Y	K	Y	NH2		
B9.4	NI*						Ac	E	A	W	D	Y	K	Y	NH2		
B9.5	NI*						Ac	E(D)	A	W	D	Y	K	Y	NH2		
B9.9	NI*					Ac	D(D)	A	A	W	D	Y	K	Y	NH2		
B9.10	NI*					D	G	A	A	W	D	Y	K	Y	NH2		
B3.14	NI*	Ac	L	V	NI*	V	G	A	A	W	D	Y	K	NH2			
B9.6	NI**				Ac	E	G	A	A	W	D	Y	K	Y	NH2		
B9.7	NI**					Ac	E	A	A	W	D	Y	K	Y	NH2		
B9.8	NI**				Ac	D(D)	G	A	A	W	D	Y	K	Y	NH2		
B2.19	NI**							Ac	A	W	D	Y	K	Y	NH2		
B3.1	NI**			Ac	NI*	V	G	A	A	W	D	Y	K	Y	NH2		
B3.8	NI**	Ac	W	V	W	V	G	A	A	W	D	Y	K	Y	NH2		
B3.10	NI**	Ac	L	V	NI*	V	G	(N-Me-Ala)	A	W	D	Y	K	(N-Me-TYR)	NH2		
B3.12	NI**				NI*	V	G	A	A	W	D	Y	K	Y			
B3.16	NI**	Ac	L	V	D	V	G	A	A	W	D	Y	K	Y	NH2		
B3.17	NI**	Ac	L	V	NI*	V	G	A	A	W	D	R	K	Y	NH2		
B3.18	NI**	Ac	L	E	NI*	K	G	A	E	W	D	Y	K	Y	NH2		
B3.2	NI**	Ac	L	V	W	V	G	A	A	W	D	Y	K	Y	NH2		
